# Development of novel anti-CDH1/E-cadherin monoclonal antibodies for versatile applications

**DOI:** 10.1016/j.bbrep.2025.102401

**Published:** 2025-12-08

**Authors:** Rena Ubukata, Hiroyuki Suzuki, Mika K. Kaneko, Yukinari Kato

**Affiliations:** Department of Antibody Drug Development, Tohoku University Graduate School of Medicine, 2-1 Seiryo-machi, Aoba-ku, Sendai, Miyagi, 980-8575, Japan

**Keywords:** E-Cadherin, CDH1, Cell-based immunization and screening, Monoclonal antibody, Flow cytometry, Immunohistochemistry

## Abstract

Cadherin (CDH)-mediated extracellular homophilic binding is crucial for maintaining tissue homeostasis. The epithelial cell-cell adhesion molecule cadherin 1 (CDH1/E‐cadherin) forms the adherens junctions in epithelial cells, and the loss of CDH1 facilitates the migration and invasion of carcinoma cells. Although several anti-CDH1 monoclonal antibodies (mAbs) are available for western blotting and immunohistochemistry (IHC), a highly sensitive anti-CDH1 mAb suitable for flow cytometry has not been developed. We developed anti-CDH1 mAbs through a flow cytometry-based high-throughput screening. Two anti-CDH1 mAb clones, Ca_1_Mab-3 (IgG_1_, κ) and Ca_1_Mab-5 (IgG_1_, κ), reacted with human CDH1-overexpressed Chinese hamster ovary-K1 (CHO/CDH1) cells in flow cytometry. Furthermore, Ca_1_Mab-3 and Ca_1_Mab-5 recognized endogenous CDH1-expressing human luminal-type breast cancer cells, such as MCF-7, but not triple-negative breast cancer cells, like MDA-MB-231. The dissociation constant values of Ca_1_Mab-3 and Ca_1_Mab-5 for CHO/CDH1 were determined as 5.9 × 10^−10^ M and 1.8 × 10^−9^ M, respectively. Ca_1_Mab-3 and Ca_1_Mab-5 can detect endogenous CDH1 in western blotting and IHC using a cell block. Furthermore, Ca_1_Mab-5 is available for IHC in formalin-fixed paraffin-embedded tumor tissues. These results indicate that Ca_1_Mab-3 and Ca_1_Mab-5 are versatile for basic research and are expected to contribute to clinical applications, such as tumor diagnosis and therapy.

## Highlights

• Novel anti-CDH1/E-cadherin monoclonal antibodies, Ca_1_Mab-3 and Ca_1_Mab-5, were developed.
• Ca_1_Mab-3 and Ca_1_Mab-5 are useful for the detection of CDH1 with high affinity by flow cytometry.
• Ca_1_Mab-3 and Ca_1_Mab-5 available for immunoblotting and immunohistochemistry using cell block.
• Ca_1_Mab-5 is also available for immunohistochemistry for tumor tissues.

## 1 Introduction

Cadherins (CDHs) are single-pass transmembrane proteins composed of extracellular and cytoplasmic regions. The extracellular portion is organized into multiple tandemly repeated units, termed extracellular cadherin (EC) domains. CDHs on the cell surface engage in homophilic binding through their N-terminal EC domains, which mediates physical cell–cell adhesion [1]. In vertebrates, the CDH family consists of approximately 20 members, which are classified into type I and type II based on subtle differences in their amino acid sequences [2]. Expression of each type I CDH is essentially cell type–specific; for instance, CDH1/E-cadherin is predominantly found in epithelial cells, CDH2/N-cadherin in neural and mesenchymal cells, CDH3/P-cadherin in placental cells, CDH4/R-cadherin in retinal cells, and CDH15/M-cadherin in muscle cells [3]. The cytoplasmic domain of CDHs is highly conserved across subtypes. It associates directly with cytoplasmic proteins such as p120-and β-catenin. β-catenin, in turn, interacts with α-catenin, which subsequently binds to F-actin, thereby establishing the CDH–actin complex [4,5].

The epithelial–mesenchymal transition (EMT) is a fundamental biological process that governs body plan formation and the differentiation of diverse tissues and organs [6]. In addition to its physiological role in tissue repair, EMT can have pathological consequences, contributing to organ fibrosis and facilitating carcinoma progression [7]. During EMT, cells lose their epithelial characteristics, such as downregulation of CDH1, and acquire invasive capabilities, gain stem cell-like properties, evade apoptosis and senescence, and promote immunosuppressive environments [7]. Consequently, the mesenchymal state is linked to enhanced cellular plasticity, enabling dissemination to distant sites, maintenance of stemness, and subsequent differentiation into multiple lineages during both development and metastatic progression [8]. The invaded tumor cells enter the blood or lymphatic vessels, thereby gaining access to the systemic circulation [8]. Among the surviving cells, some can arrest within a distant capillary and extravasate into a secondary organ. Upon colonization of distant tissues, these mesenchymal-like cells may undergo a reverse process, the mesenchymal–epithelial transition (MET), which restores proliferative capacity and facilitates the formation of macroscopic secondary tumors [6,9].

Anti-CDH1 monoclonal antibodies (mAbs) have been developed for various applications. A clone ECCD-1 (E-cadherin Cell-Cell adhesion Disrupting-1) recognizes CDH1 ectodomain and could block CDH1-mediated cell aggregation [10]. Another clone, DECMA-1, recognizes the CDH1 ectodomain and can be used in various applications, including immunoprecipitation, western blotting, immunofluorescence, and biological functions (inhibition of cell aggregation) [11]. DECMA-1 is also used in studies targeting CDH1 in tumor xenograft models [12]. Several anti-CDH1 mAbs against the cytoplasmic domain exhibited a superior reactivity in immunohistochemistry (IHC) [13]. However, few mAbs have been developed for flow cytometry.

We previously established a series of mAbs targeting membrane proteins, including CDH15 [14], chemokine receptors [15], and receptor tyrosine kinases [16,17] using the Cell-Based Immunization and Screening (CBIS) method. The CBIS method involves immunization with antigen-overexpressed cells, followed by high-throughput flow cytometry–based screening. As a result, mAbs generated through the CBIS method typically recognize conformational epitopes and are particularly suitable for flow cytometric analysis. Additionally, several of these mAbs have been demonstrated to be suitable for Western blotting and IHC. In the present study, we employed the CBIS method to develop highly versatile anti-CDH1 mAbs.

## 2 Materials and methods

### 2.1 Cell lines

Chinese hamster ovary (CHO)–K1, mouse myeloma P3X63Ag8U.1 (P3U1), human glioblastoma LN229, and human breast cancer MDA-MB-231 cell lines were obtained from American Type Culture Collection (ATCC, Manassas, VA, USA). Human breast cancer MCF-7 was obtained from the Cell Resource Center for Biomedical Research Institute of Development, Aging, and Cancer at Tohoku University (Miyagi, Japan). Human embryonic kidney 293FT was purchased from Thermo Fisher Scientific, Inc. (Waltham, MA, USA).

LN229, 293FT, MCF-7, and MDA-MB-231 were maintained in Dulbecco's Modified Eagle Medium (Nacalai Tesque, Inc., Kyoto, Japan), supplied with 100 U/mL penicillin, 100 μg/mL streptomycin, 0.25 μg/mL amphotericin B (Nacalai Tesque, Inc.), and 10 % heat-inactivated fetal bovine serum (FBS; Thermo Fisher Scientific, Inc.). CHO–K1 and P3U1 were maintained in Roswell Park Memorial Institute-1640 medium (Nacalai Tesque, Inc.), supplied with 100 U/mL penicillin, 100 μg/mL streptomycin, 0.25 μg/mL amphotericin B, and 10 % heat-inactivated FBS. All the cells were cultured in a humidified incubator at 37 °C with 5 % CO_2_.

### 2.2 Plasmid construction and establishment of stable transfectants

We previously established the type I CDH-overexpressed stable transfectants: CHO/CDH1, CHO/PA16-CDH2 (CHO/CDH2), CHO/CDH3, CHO/PA16-CDH4 (CHO/CDH4), and CHO/PA16-CDH15 (CHO/CDH15) [14]. The N-terminal PA16-tag (GLEGGVAMPGAEDDVV) was recognized by an anti-PA16 tag mAb, NZ-1 [18].

The pCMV6neo-CDH1-myc-DDK (OriGene Technologies, Inc., Rockville, MD, USA) vector was transfected into LN229 using the Neon transfection system (Thermo Fisher Scientific, Inc.). The transfectants were sorted using an anti-CDH1 mAb (clone DECMA-1, BioLegend, San Diego, CA, USA) with an SH800 cell sorter (Sony Corporation, Tokyo, Japan). The CDH1-overexpressed LN229 (LN229/CDH1) was maintained in a medium containing 0.5 mg/mL of G418 (Nacalai Tesque, Inc.).

### 2.3 Hybridoma production

Female BALB/cAJcl mice were obtained from CLEA Japan (Tokyo, Japan). All animal experiments were approved by the Animal Care and Use Committee of Tohoku University (Permit No. 2022MdA-001) and conducted in accordance with the NIH Guide for the Care and Use of Laboratory Animals. Mice were intraperitoneally immunized with LN229/CDH1 cells (1 × 10^8^ cells/injection) emulsified with 2 % Alhydrogel adjuvant (InvivoGen). Following three additional weekly immunizations (1.0 or 1.5 × 10^8^ cells/injection), a booster dose (1 × 10^8^ cells/injection) was administered two days prior to spleen excision. Hybridomas were generated as previously described [19]. Supernatants positive for CHO/CDH1 and negative for CHO–K1 were screened using an SA3800 Cell Analyzer (Sony Corporation, Tokyo, Japan).

### 2.4 Flow cytometry

Another commercially available anti-CDH1 mAb (clone 67A4) was purchased from BD Biosciences (Franklin Lakes, NJ, USA). The recommended applications are flow cytometry, Western blot, and immunofluorescence. An isotype control mAb (CvMab-62, IgG_1_, κ) was previously described [20]. Cells were harvested with 1 mM ethylenediaminetetraacetic acid (EDTA; Nacalai Tesque, Inc.). A total of 1 × 10^5^ cells were washed with phosphate-buffered saline (PBS) containing 0.1 % bovine serum albumin (BSA; blocking buffer) and incubated with mAbs for 30 min at 4 °C. Subsequently, the cells were stained with Alexa Fluor 488-conjugated anti-mouse IgG or anti-rat IgG (2000-fold dilution; Cell Signaling Technology, Danvers, MA, USA) for 30 min at 4 °C. Flow cytometric data were acquired on an SA3800 Cell Analyzer by collecting 5000 events. Cells were gated based on forward scatter (FSC) and side scatter (SSC), and fluorescence intensity was analyzed using FlowJo software (BD Biosciences).

### 2.5 Determination of dissociation constant values using flow cytometry

MCF-7 and CHO/CDH1 were treated with serially diluted Ca_1_Mab-3 and Ca_1_Mab-5. Subsequently, the cells were treated with anti-mouse IgG conjugated with Alexa Fluor 488 (200-fold dilution) for 30 min at 4 °C. The data (10,000 events) were collected using BD FACSLyric (BD Biosciences), and the geometric mean (GeoMean) was determined using FlowJo. The fitting binding isotherms (vertical axis, GeoMean; horizontal axis, mAb concentration) determined the dissociation constant (*K*_D_) values to built-in one-side binding models of GraphPad Prism 6 (GraphPad Software, Inc., La Jolla, CA, USA).

### 2.6 Western blotting

Whole-cell lysates (10 μg of protein) were separated into polyacrylamide gels and transferred onto polyvinylidene difluoride membranes (Merck KGaA, Darmstadt, Germany). The membranes were blocked with 4 % skim milk (Nacalai Tesque, Inc.) in PBS containing 0.05 % Tween 20 and incubated with 5 μg/mL of Ca_1_Mab-3, 5 μg/mL of Ca_1_Mab-5, or 1 μg/mL of an anti-isocitrate dehydrogenase 1 (IDH1) mAb (clone RcMab-1, rat IgG_2a_). Then, the membranes were incubated with anti-mouse IgG (1000-fold dilution; Agilent Technologies, Inc., Santa Clara, CA, USA) or anti-rat IgG conjugated with horseradish peroxidase (10,000-fold dilution; Sigma-Aldrich Corp., St. Louis, MO, USA). Chemiluminescence signals were developed and detected as described previously [21].

### 2.7 Immunohistochemistry (IHC) using cell blocks

Cells were fixed with 4 % paraformaldehyde, and the cell blocks were prepared using iPGell (Genostaff Co., Ltd., Tokyo, Japan) (FUJIFILM Wako Pure Chemical Corporation). The formalin-fixed paraffin-embedded (FFPE) cell sections were stained with Ca_1_Mab-3 (1 μg/mL) or Ca_1_Mab-5 (1 μg/mL) using BenchMark ULTRA PLUS with the OptiView DAB IHC Detection Kit (Roche Diagnostics, Indianapolis, IN, USA).

### 2.8 Immunohistochemical analysis (IHC)

The FFPE oral squamous cell carcinoma (OSCC) tissue array (OR601c) and breast invasive ductal carcinoma tissue array (T088b) were purchased from US Biomax Inc. (Rockville, MD, USA). The sections were stained with Ca_1_Mab-3 (10 μg/mL) or Ca_1_Mab-5 (10, or 25 μg/mL) as described in 2.7.

## 3 Results

### 3.1 Development of anti-CDH1 mAbs

**Fig. 1:**
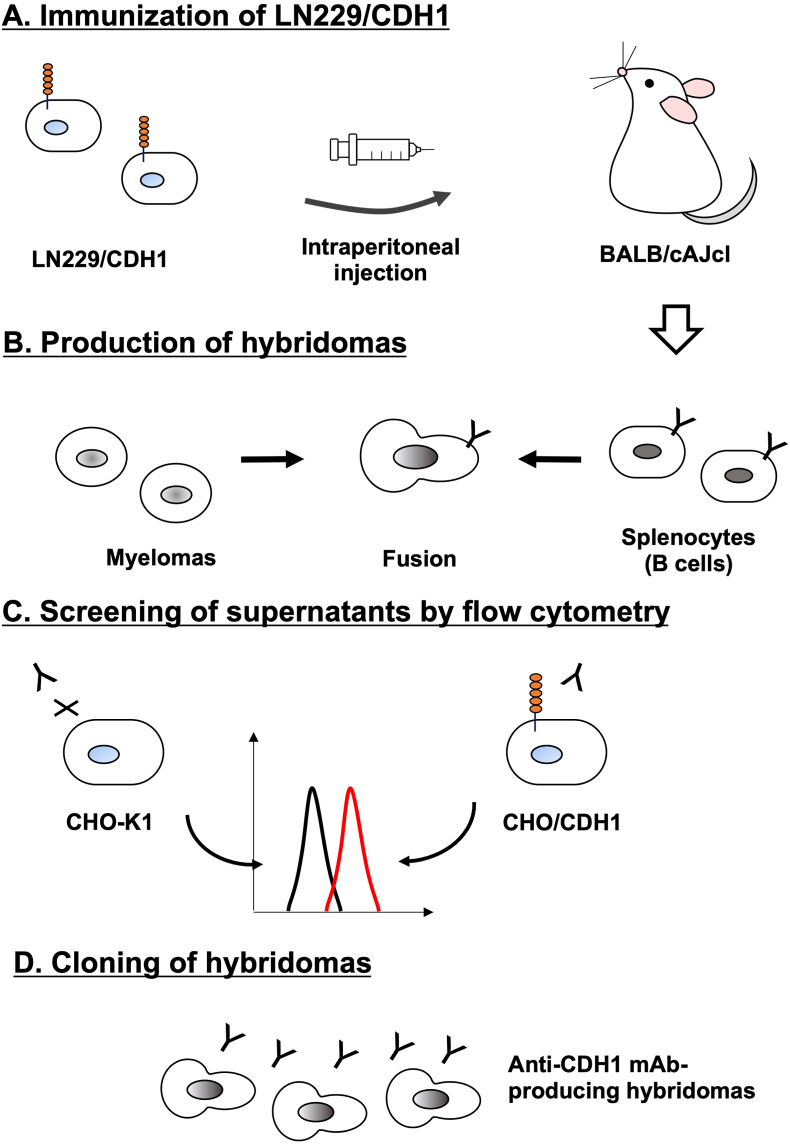
Schematic representation of anti-CDH1 mAbs production. (A) LN229/CDH1 was injected intraperitoneally into BALB/cAJcl mice. (B) After five immunizations per week, spleen cells were fused with P3U1. (C) The supernatants from hybridomas were screened by flow cytometry using CHO/CDH1 and CHO–K1 cells. (D) Anti-CDH1 mAb-producing hybridoma clones (Ca_1_Mabs) were established through limiting dilution. LN229/CDH1, CDH1-overexpressed LN229; CHO, Chinese hamster ovary; CHO/CDH1, CDH1-overexpressed CHO–K1.

**Supplementary Fig. 1:**
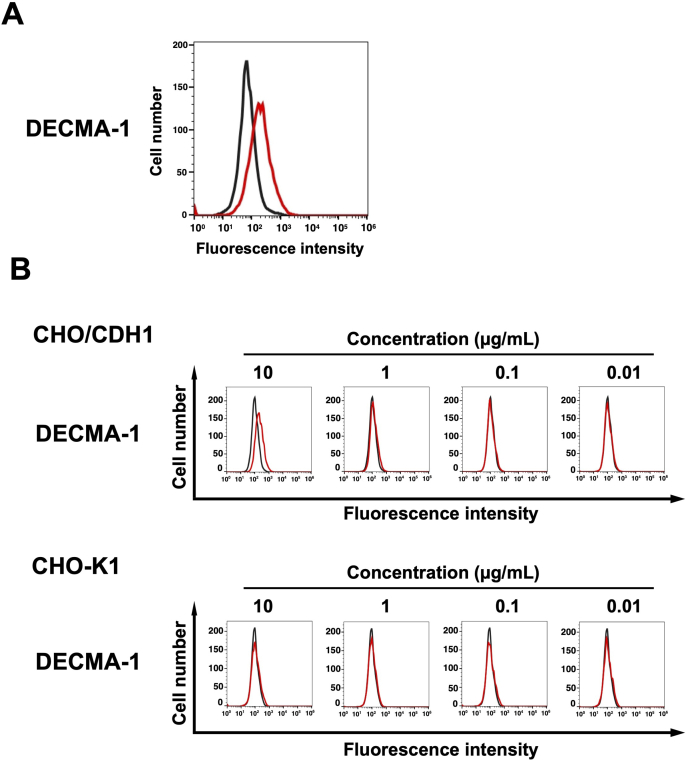
Flow cytometry analysis of an anti-CDH1 mAb, DECMA-1 against LN229/CDH1, LN229, CHO/CDH1, and CHO–K1. (A) LN229/CDH1 (red) and LN229 (black) were treated with 10 μg/mL of DECMA-1. (B) CHO/CDH1 and CHO–K1 were treated with DECMA-1 at the indicated concentrations (red) or with blocking buffer (black, negative control). The mAb-treated cells were incubated with anti-rat IgG conjugated with Alexa Fluor 488. The fluorescence data were collected using the SA3800 Cell Analyzer.

LN229/CDH1 was established as an antigen by sorting using an anti-CDH1 mAb, DECMA-1. Although the reactivity of DECMA-1 in flow cytometry was low (Supplementary Fig. 1A), LN229/CDH1 was immunized in two BALB/cAJcl mice for five times (Fig. 1A). Then, hybridomas were generated (Fig. 1B), and the supernatants were screened by a flow cytometry-based high-throughput screening to identify supernatants that were negative for CHO–K1 and positive for CHO/CDH1 (Fig. 1C). As a result, 61 positive wells out of 956 wells (6.4 %) were obtained. Subsequently, limiting dilution was performed and anti-CDH1 mAb-producing hybridoma clones were established (Fig. 1D). Finally, clones Ca_1_Mab-1 (IgG_2a_, κ), Ca_1_Mab-2 (IgM, κ), Ca_1_Mab-3 (IgG_1_, κ), Ca_1_Mab-5 (IgG_1_, κ), Ca_1_Mab-6 (IgM, κ), Ca_1_Mab-7 (IgG_1_, κ), Ca_1_Mab-8 (IgM, κ), and Ca_1_Mab-9 (IgM, κ) were established.

### 3.2 Flow cytometry using Ca_1_Mab-3 and Ca_1_Mab-5

**Fig. 2:**
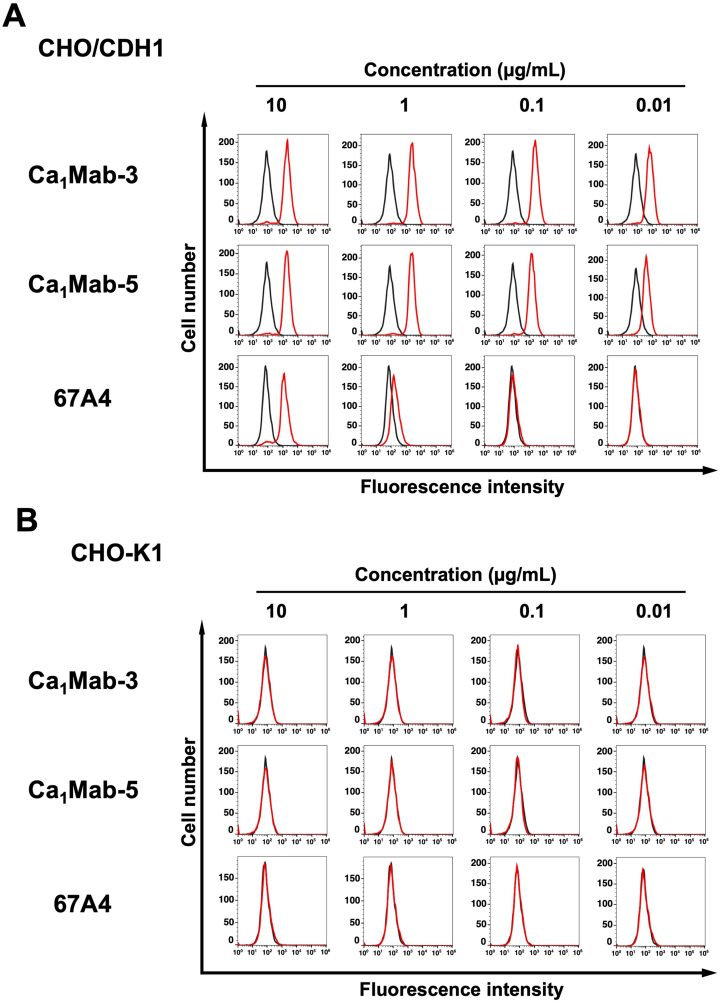
Flow cytometry analysis of anti-CDH1 mAbs, Ca_1_Mab-3, Ca_1_Mab-5, and 67A4 against CHO/CDH1 and CHO–K1. CHO/CDH1 (A) and CHO–K1 (B) were treated with Ca_1_Mab-3, Ca_1_Mab-5, and 67A4 at the indicated concentrations (red) or with blocking buffer (black, negative control). The mAbs-treated cells were incubated with anti-mouse IgG conjugated with Alexa Fluor 488. The fluorescence data were collected using the SA3800 Cell Analyzer.

Using the supernatants of clones, we next conducted the screening of applications, including flow cytometry, western blotting, and IHC using the CHO/CDH1 cell block (http://www.med-tohoku-antibody.com/topics/001_paper_antibody_PDIS.htm#CDH1+). Since Ca_1_Mab-3 and Ca_1_Mab-5 can be applied to the three applications, we further investigated the properties of these mAbs. Fig. 2 shows flow cytometry analysis using the Ca_1_Mab-3 and Ca_1_Mab-5 against CHO/CDH1 and CHO–K1 cells. Ca_1_Mab-3 and Ca_1_Mab-5 recognized CHO/CDH1 in a dose-dependent manner (Fig. 2A) from 10 to 0.01 μg/mL, but did not recognize CHO–K1 even at 10 μg/mL (Fig. 2B). Since DECMA-1 also showed low reactivity to CHO/CDH1 (Supplementary Fig. 1B), another commercially available anti-CDH1 mAb, 67A4, was used. Compared to 67A4, Ca_1_Mab-3 and Ca_1_Mab-5 clearly showed a higher reactivity to CHO/CDH1 (Fig. 2A).

**Fig. 3:**
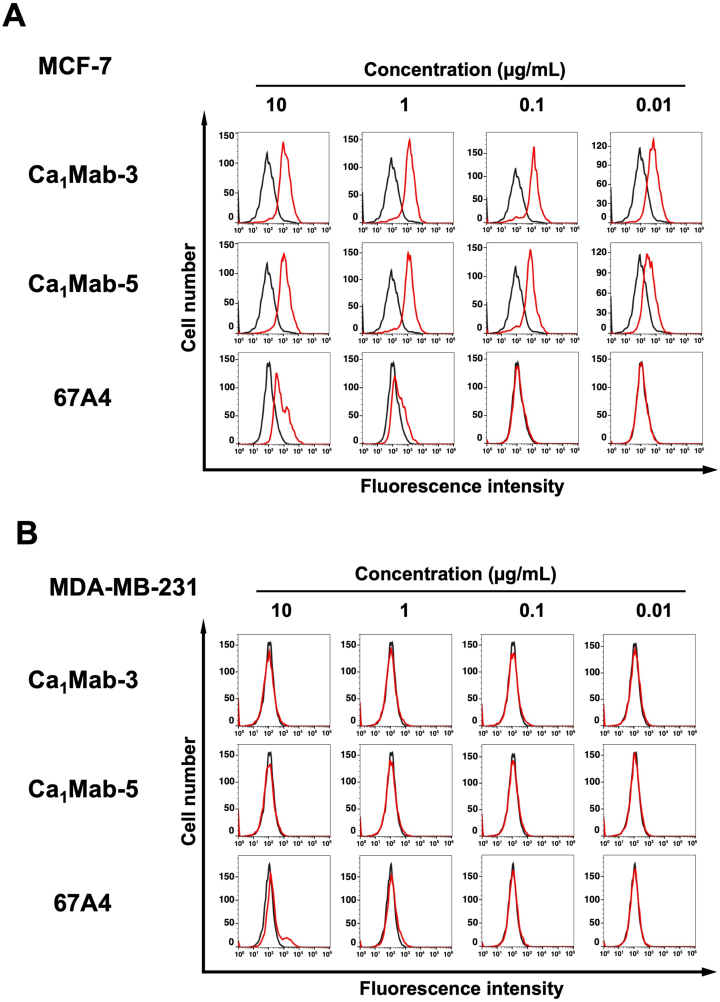
Flow cytometry analysis of Ca_1_Mab-3, Ca_1_Mab-5, and 67A4 against MCF-7 and MDA-MB-231. MCF-7 (A) and MDA-MB-231 (B) were treated with Ca_1_Mab-3, Ca_1_Mab-5, and 67A4 at the indicated concentrations (red) or with blocking buffer (black, negative control). The mAbs-treated cells were incubated with anti-mouse IgG conjugated with Alexa Fluor 488. The fluorescence data were collected using the SA3800 Cell Analyzer.

**Supplementary Fig. 2:**
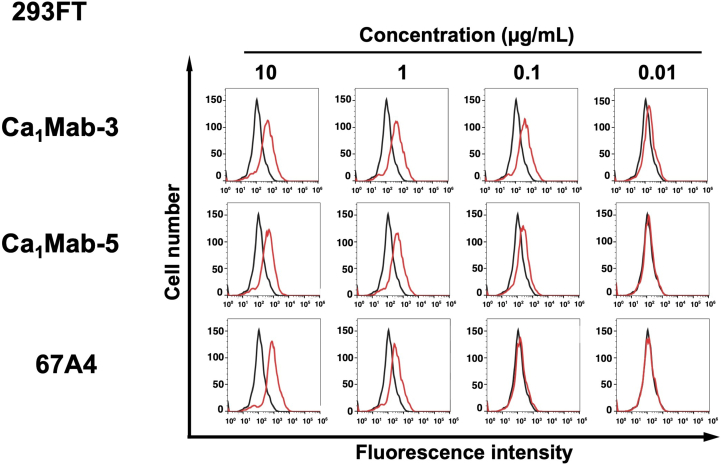
Flow cytometry analysis of Ca_1_Mab-3, Ca_1_Mab-5, and 67A4 against 293FT. 293FT were treated with Ca_1_Mab-3, Ca_1_Mab-5, and 67A4 at the indicated concentrations (red) or with blocking buffer (black, negative control). The mAbs-treated cells were incubated with anti-mouse IgG conjugated with Alexa Fluor 488. The fluorescence data were collected using the SA3800 Cell Analyzer.

**Supplementary Fig. 3:**
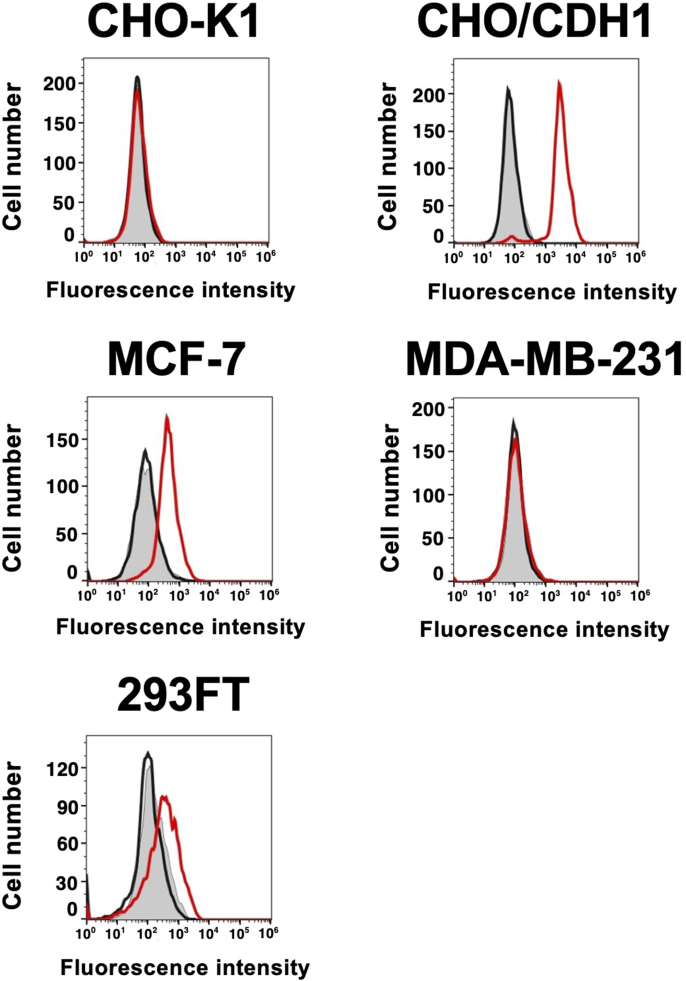
Flow cytometry analysis using isotype control mAb. Cells were treated with 10 μg/mL of CvMab-62, an isotype control IgG_1_ mAb (filled gray), 10 μg/mL of Ca_1_Mab-3 (red), or blocking buffer (black, negative control). The mAbs-treated cells were incubated with anti-mouse IgG conjugated with Alexa Fluor 488. The fluorescence data were collected using the SA3800 Cell Analyzer.

Previous studies have showed that CDH1 expression is observed in luminal-type breast cancers, such as MCF-7, but not in basal B-type breast cancers, including MDA-MB-231 [22]. Ca_1_Mab-3 and Ca_1_Mab-5 recognized MCF-7 and showed a higher reactivity compared to 67A4 (Fig. 3A). Ca_1_Mab-3 and Ca_1_Mab-5 did not recognize MDA-MB-231 even at 10 μg/mL, but weak reactivity of 67A4 to MDA-MB-231 was observed at 10 μg/mL (Fig. 3B). We also found that Ca_1_Mab-3 and Ca_1_Mab-5 reacted to epithelial cell lines, including 293FT (Supplementary Fig. 2). We also confirmed that isotype control mAb (CvMab-62) did not recognize cell lines used in this study even at 10 μg/mL (Supplementary Fig. 3). These results indicate that Ca_1_Mab-3 and Ca_1_Mab-5 can be applied to flow cytometry for the detection of both exogenous and endogenous CDH1.

### 3.3 Specificity of Ca_1_Mab-3 and Ca_1_Mab-5 to type I CDHs-overexpressed CHO–K1

**Fig. 4:**
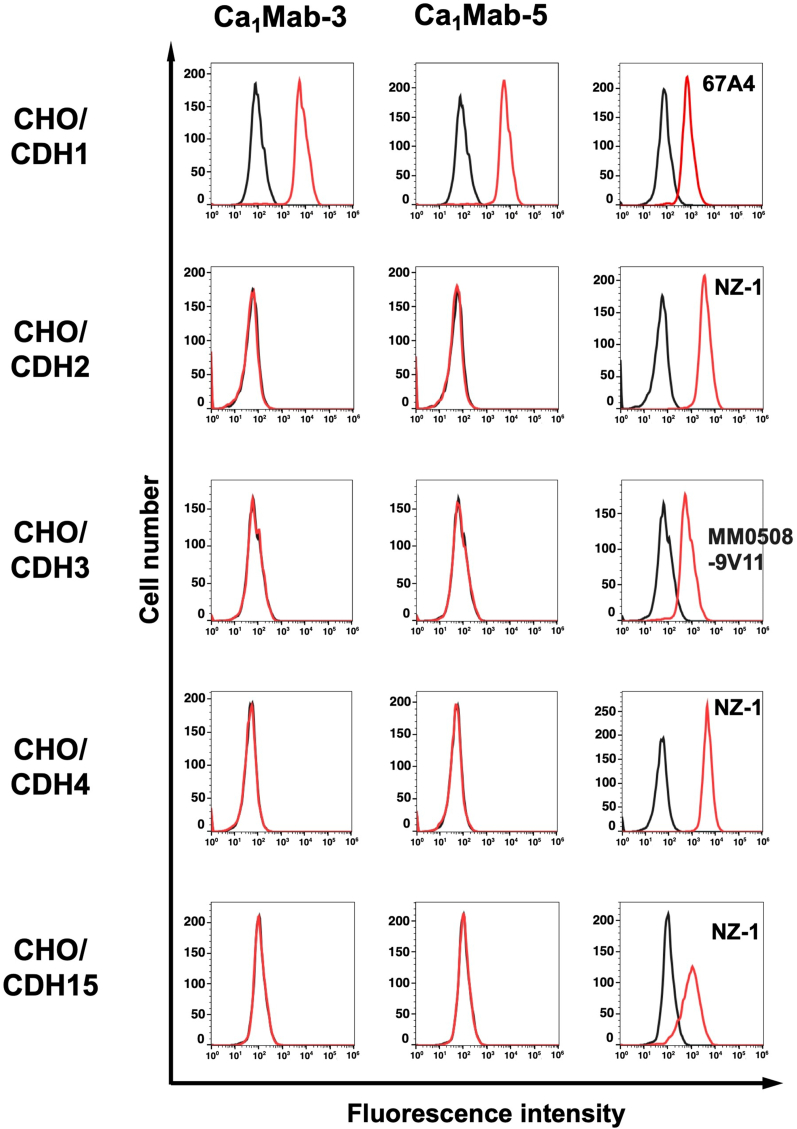
Flow cytometry analysis of Ca_1_Mab-3 and Ca_1_Mab-5 in type I CDHs-overexpressed CHO–K1. The type I CDHs (CDH1, CDH2, CDH3, CDH4, and CDH15)-expressed CHO–K1 cells were treated with 10 μg/mL of Ca_1_Mab-3 and Ca_1_Mab-5 (red) or with control blocking buffer (black, negative control), followed by treatment with anti-mouse IgG conjugated with Alexa Fluor 488. Each CDH expression was confirmed by 10 μg/mL of an anti-CDH1 mAb (clone 67A4), 1 μg/mL of an anti-CDH3 mAb (clone MM0508-9V11), and 1 μg/mL of an anti-PA16-tag mAb (clone NZ-1) to detect PA16-tagged CDH2, CDH4, and CDH15, followed by the treatment with anti-mouse IgG or anti-rat IgG conjugated with Alexa Fluor 488. The fluorescence data were collected using the SA3800 Cell Analyzer.

**Supplementary Fig. 4:**
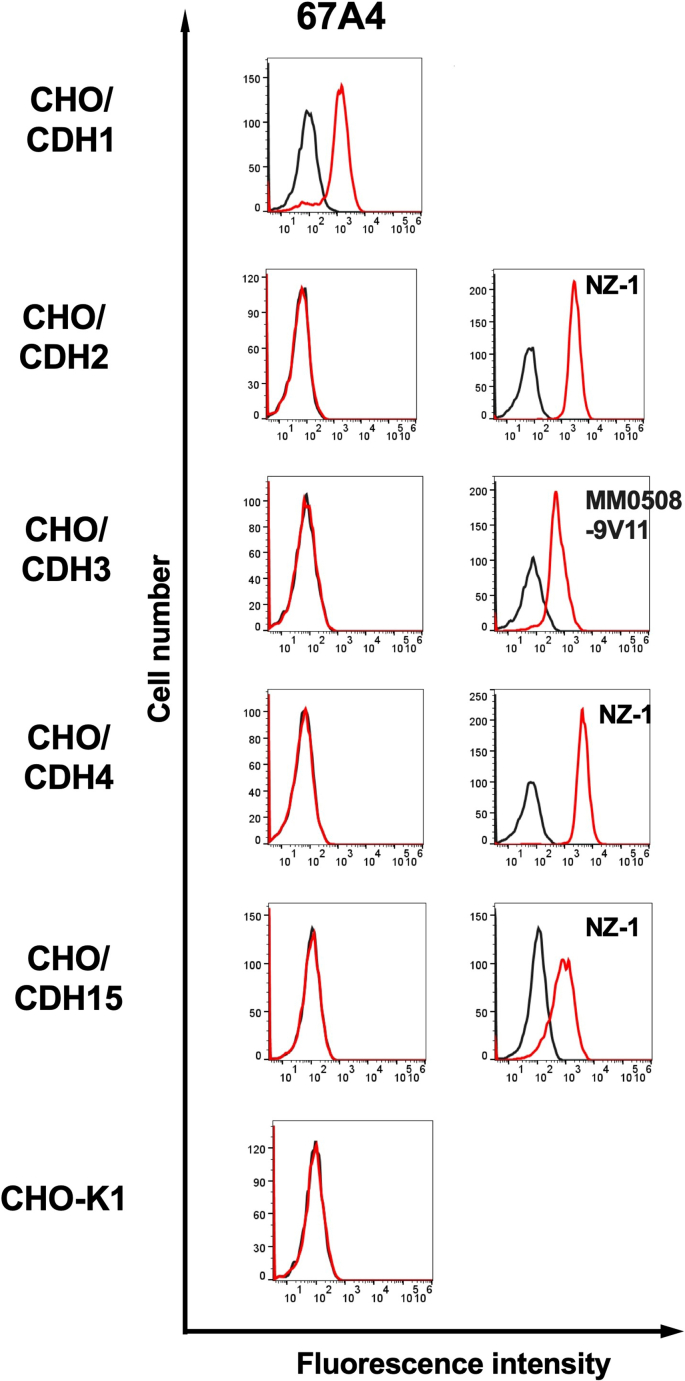
Flow cytometry analysis of 67A4 in type I CDHs-overexpressed CHO–K1. The type I CDHs (CDH1, CDH2, CDH3, CDH4, and CDH15)-expressed CHO–K1 cells were treated with 10 μg/mL of 67A4 (red) or with control blocking buffer (black, negative control), followed by treatment with anti-mouse IgG conjugated with Alexa Fluor 488. Each CDH expression was confirmed by 1 μg/mL of an anti-CDH3 mAb (clone MM0508-9V11), and 1 μg/mL of an anti-PA16-tag mAb (clone NZ-1) to detect PA16-tagged CDH2, CDH4, and CDH15, followed by the treatment with anti-mouse IgG or anti-rat IgG conjugated with Alexa Fluor 488. The fluorescence data were collected using the SA3800 Cell Analyzer.

The type I CDH includes CDH1/E-cadherin, CDH2/N-cadherin, CDH3/P-cadherin, CDH4/R-cadherin, and CDH15/M-cadherin [3]. We previously established CHO–K1, which expresses each type I CDH [14]. Therefore, the specificity of Ca_1_Mab-3 and Ca_1_Mab-5 to type I CDHs was determined. As shown in Fig. 4, Ca_1_Mab-3 and Ca_1_Mab-5 reacted with CHO/CDH1 but did not react with other type I CDHs-overexpressed CHO–K1. 67A4 also exhibited the specificity to CHO/CDH1 (Supplementary Fig. 4). These results indicate that Ca_1_Mab-3 and Ca_1_Mab-5 are specific mAbs against CDH1 among type I CDHs.

### *3.4* *Determination of K*_D_*values of Ca*_*1*_*Mab-3 and Ca*_*1*_*Mab-*5 by *flow cytometry*

**Fig. 5:**
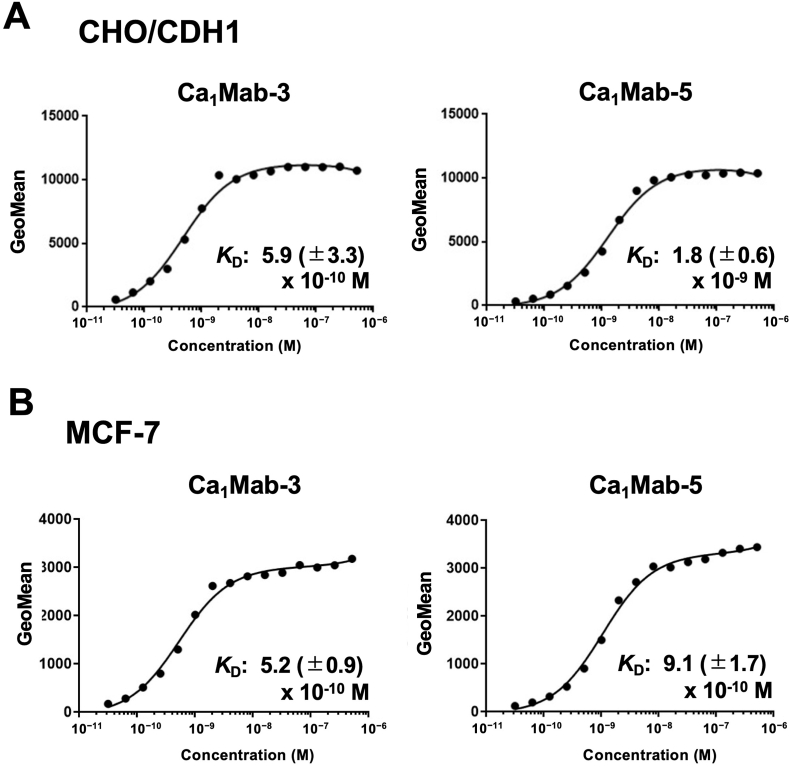
Measurement of binding affinity of Ca_1_Mab-3 and Ca_1_Mab-5. CHO/CDH1 (A) and MCF-7 (B) were treated with serially diluted Ca_1_Mab-3 or Ca_1_Mab-5, followed by anti-mouse IgG conjugated with Alexa Fluor 488. The fluorescence data were analyzed using the BD FACSLyric. The *K*_D_ values were determined using GraphPad PRISM 6. The average *K*_D_ values (± standard deviation) from three independent measurements were calculated by GraphPad PRISM 6 software. The representative graphs were shown.

**Supplementary Fig. 5:**
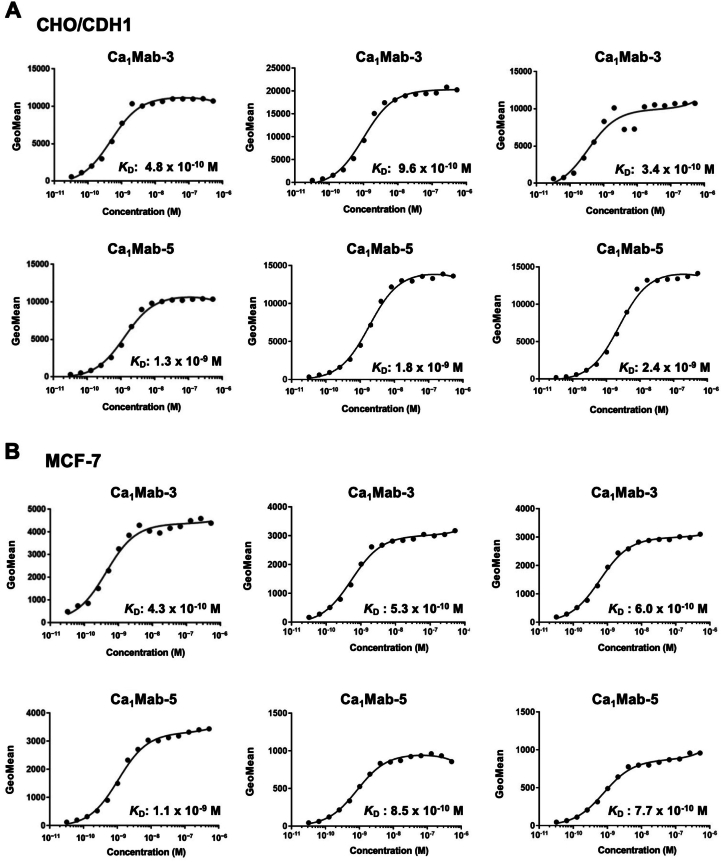
Three independent measurements of the binding affinity of Ca_1_Mab-3 and Ca_1_Mab-5. CHO/CDH1 (A) and MCF-7 (B) were treated with serially diluted Ca_1_Mab-3 or Ca_1_Mab-5, followed by anti-mouse IgG conjugated with Alexa Fluor 488. The fluorescence data were analyzed using the BD FACSLyric. The *K*_D_ values were determined using GraphPad PRISM 6.

The binding affinity of Ca_1_Mab-3 and Ca_1_Mab-5 was measured using flow cytometry. The fitting binding isotherms of Ca_1_Mab-3 and Ca_1_Mab-5 to CHO/CDH1 and MCF-7 were shown in Fig. 5. The three replicates were shown in Supplementary Fig. 5. The *K*_D_ values of Ca_1_Mab-3 for CHO/CDH1 and MCF-7 were (5.9 ± 3.3) × 10^−10^ M and (5.2 ± 0.9) × 10^−10^ M, respectively. The *K*_D_ values of Ca_1_Mab-5 for CHO/CDH1 and MCF-7 were (1.8 ± 0.6) × 10^−9^ M and (9.1 ± 1.7) × 10^−10^ M, respectively. These results showed that Ca_1_Mab-3 possesses superior binding affinity to CDH1-positive cells compared to Ca_1_Mab-5.

### 3.5 Western blot analysis using Ca_1_Mab-3 and Ca_1_Mab-5

**Fig. 6:**
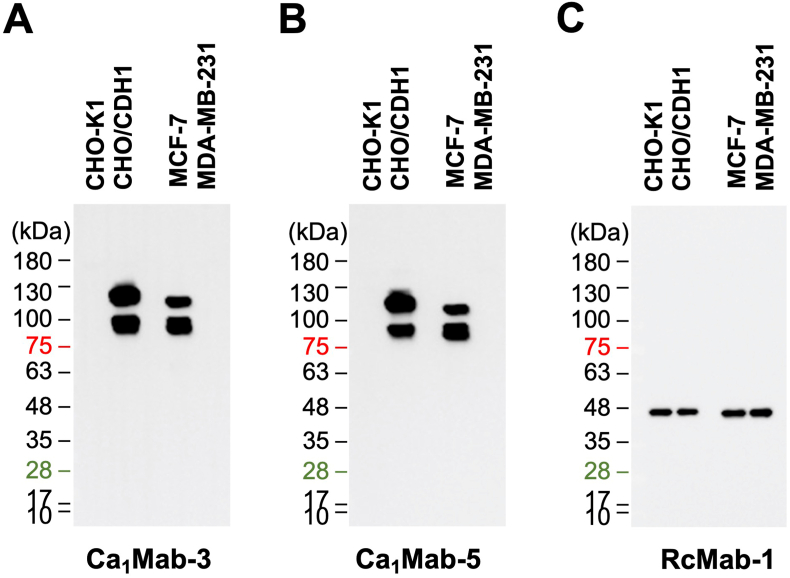
Western blotting using Ca_1_Mab-3 and Ca_1_Mab-5. The cell lysate (10 μg/lane) of CHO–K1, CHO/CDH1, MCF-7, and MDA-MB-231 were electrophoresed and transferred onto polyvinylidene difluoride membranes. The membranes were incubated with 5 μg/mL of Ca_1_Mab-3 (A), 5 μg/mL of Ca_1_Mab-5 (B), and 1 μg/mL of RcMab-1 (an anti-IDH1 mAb) (C), followed by the treatment with anti-mouse (Ca_1_Mab-3 and Ca_1_Mab-5) or anti-rat IgG (RcMab-1)-conjugated with horseradish peroxidase.

We next assessed whether Ca_1_Mab-3 and Ca_1_Mab-5 are suitable for western blotting. Whole-cell lysates of CHO–K1, CHO/CDH1, MCF-7, and MDA-MB-231 were analyzed. Ca_1_Mab-3 and Ca_1_Mab-5 exhibited clear bands from 100 to 130 kDa in CHO/CDH1 and MCF-7, but not in CHO–K1 and MDA-MB-231 (Fig. 6A and B). An anti-IDH1 mAb (RcMab-1) served as an internal control (Fig. 6C). These results indicate that Ca_1_Mab-3 and Ca_1_Mab-5 can detect exogenous and endogenous CDH1 in western blotting.

### 3.6 IHC using Ca_1_Mab-3 and Ca_1_Mab-5 in formalin-fixed paraffin-embedded cell blocks

**Fig. 7:**
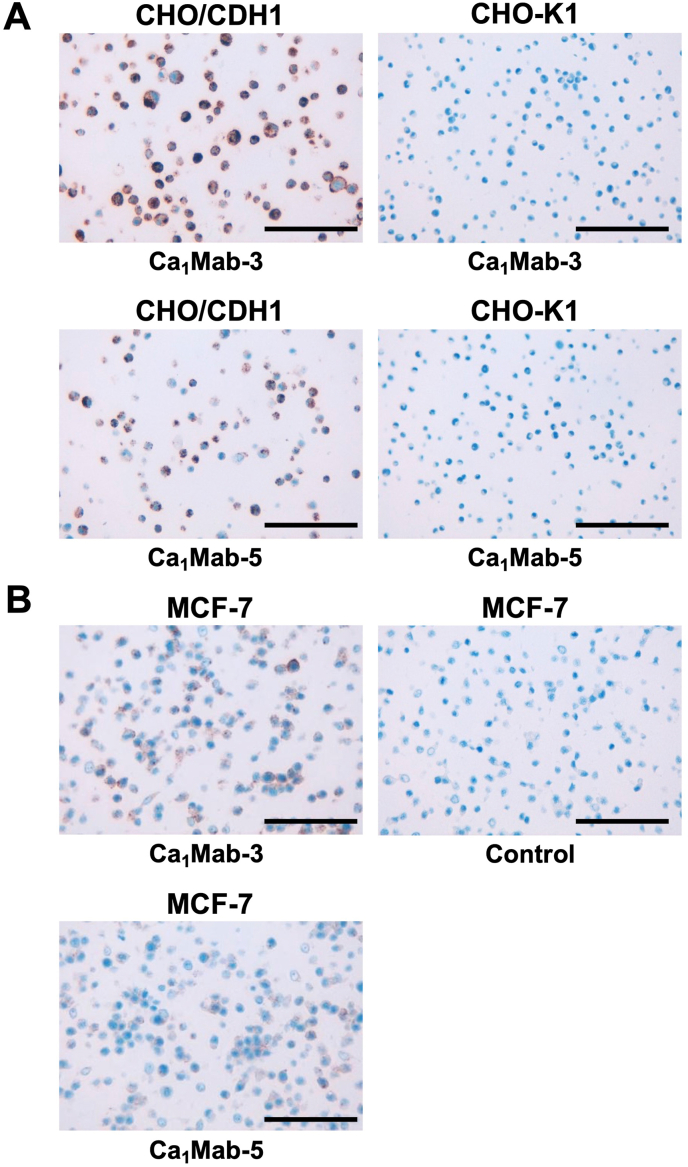
Immunohistochemistry using Ca_1_Mab-3 and Ca_1_Mab-5 in formalin-fixed paraffin-embedded cell blocks. (A) CHO/CDH1 and CHO–K1 sections were treated with 1 μg/mL of Ca_1_Mab-3 or Ca_1_Mab-5. (B) MCF-7 sections were treated with 1 μg/mL of Ca_1_Mab-3, 1 μg/mL of Ca_1_Mab-5, or control (without primary Ab). The staining was performed using BenchMark ULTRA PLUS with the OptiView DAB IHC Detection Kit. Scale bar = 100 μm.

We examined whether Ca_1_Mab-3 and Ca_1_Mab-5 are suitable for the IHC of FFPE sections of CHO–K1, CHO/CDH1, and MCF-7. Both intense cytoplasmic and membranous staining by Ca_1_Mab-3 were detected in CHO/CDH1 but not in CHO–K1 (Fig. 7A). Using Ca_1_Mab-5, a weaker staining pattern was observed (Fig. 7A). Furthermore, cytoplasmic and membranous staining by Ca_1_Mab-3 and Ca_1_Mab-5 were observed in MCF-7 (Fig. 7B). These results indicate that Ca_1_Mab-3 and Ca_1_Mab-5 can detect exogenous and endogenous CDH1 in IHC of FFPE sections of cultured cells.

### 3.7 IHC using Ca_1_Mab-3 and Ca_1_Mab-5 in formalin-fixed paraffin-embedded tissues

**Fig. 8:**
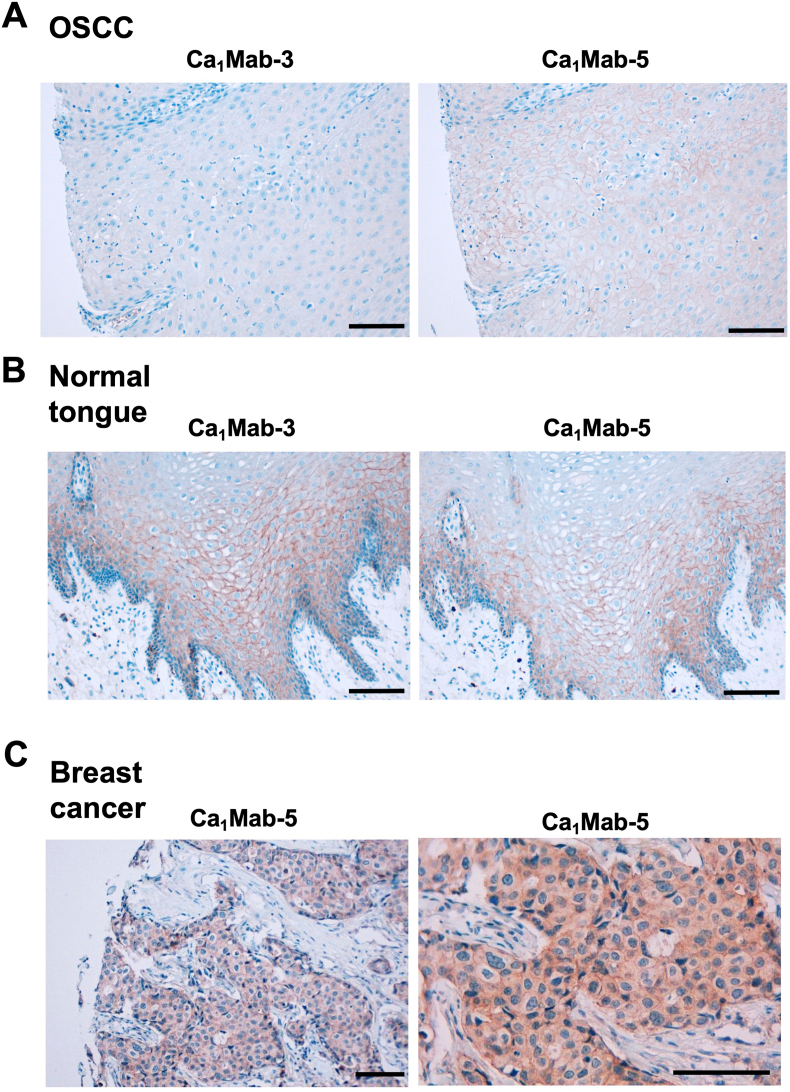
Immunohistochemistry using Ca_1_Mab-3 and Ca_1_Mab-5 in formalin-fixed paraffin-embedded tissues. (A) The sequential sections of a FFPE OSCC tissue were stained with 10 μg/mL of Ca_1_Mab-3 and Ca_1_Mab-5. (B) The sequential sections of a normal tongue squamous epithelium were stained with 10 μg/mL of Ca_1_Mab-3 and Ca_1_Mab-5. (C) A FFPE breast invasive ductal carcinoma tissue was stained with 25 μg/mL of Ca_1_Mab-5. The staining was performed using BenchMark ULTRA PLUS with the OptiView DAB IHC Detection Kit. Scale bar = 100 μm.

The FFPE OSCC tissue array was stained with Ca_1_Mab-3 and Ca_1_Mab-5. Ca_1_Mab-5, but not Ca_1_Mab-3, exhibited a membranous staining in OSCC sections (Fig. 8A). In contrast, both Ca_1_Mab-5 and Ca_1_Mab-3 showed a membranous staining in normal tongue squamous epithelium (Fig. 8B). Furthermore, Fig. 8C showed the cytoplasmic and membranous staining of a breast invasive ductal carcinoma tissue using Ca_1_Mab-5. These results indicated that Ca_1_Mab-5 is suitable for detecting CDH1 in FFPE tumor sections.

## 4 Discussion

This study reported novel anti-CDH1 mAbs for flow cytometry using the CBIS method (Fig. 1). Two anti-CDH1 mAbs, Ca_1_Mab-3 and Ca_1_Mab-5, recognized both exogenous and endogenous CDH1 in flow cytometry with high reactivity compared to commercially available anti-CDH1 mAbs (DECMA-1 and 67A4) (Fig. 2, Fig. 3, and Supplementary Fig. 1) and a superior affinity (Fig. 5). Ca_1_Mab-3 and Ca_1_Mab-5 showed the specificity among type I CDHs (Fig. 4). Furthermore, Ca_1_Mab-3 and Ca_1_Mab-5 are suitable for western blotting (Fig. 6) and IHC using cell block (Fig. 7). Additionally, Ca_1_Mab-5 could stain the cell-cell junction in IHC using FFPE tumor tissues (Fig. 8). Since IHC were performed using an automated slide staining system, BenchMark ULTRA PLUS, it is possible to standardize the staining conditions. Therefore, Ca_1_Mab-3 and Ca_1_Mab-5 are highly versatile for basic research and are expected to contribute to the understanding of epithelial and tumor biology.

Since Ca_1_Mab-3 can recognize a single cell by flow cytometry with high affinity (Fig. 2, Fig. 3, Fig. 5) or IHC using a cell block (Fig. 7), Ca_1_Mab-3 is suitable for detecting or isolating individual epithelial or tumor cells. In the fluorescence-activated cell sorting system, Ca_1_Mab-3 will be useful for isolating CDH1-positive cells or performing negative selection. In IHC of tumor sections, high concentrations (10 or 25 μg/mL) of Ca_1_Mab-3 or Ca_1_Mab-5 were required for the detection (Fig. 8). The structure of CDH1 is changed from closed monomer to strand-swap dimer when it forms adherens junction [23]. Therefore, Ca_1_Mab-3 and Ca_1_Mab-5 preferentially recognize the closed monomer conformation but have difficulty recognizing the strand-swap dimer conformation. Additionally, it is unclear why Ca_1_Mab-3 recognizes only normal squamous epithelial tissue but not tumor tissue (Fig. 8). We will clarify this difference by determining the epitopes of Ca_1_Mab-3 and Ca_1_Mab-5.

Circulating tumor cells (CTCs) enter the peripheral circulation from primary or metastatic lesions either spontaneously or as a result of therapeutic manipulation [24]. Some CTCs may evade from immune systems, ultimately resulting in the establishment of microscopic cancer foci and metastasis [25]. CTCs have been identified across diverse cancer types using panels of molecular markers. Epithelial cell adhesion molecule (EpCAM) is the most extensively utilized marker [26]. The level of EpCAM expression varies among cancer types, and EpCAM-based CTC detection systems are particularly effective in malignancies with high EpCAM expression, such as prostate and breast cancers [27,28]. In addition, other epithelial-derived malignancies including colorectal [29], pancreatic [30], and hepatocellular carcinomas [31] also exhibit substantial frequencies of EpCAM-positive CTCs. The detection of EpCAM-positive CTCs is consistently associated with early distant metastasis and unfavorable patient survival outcomes [19,32]. Studies have demonstrated that E-cadherin is expressed at higher levels than EpCAM on clustered CTCs which exhibit potent metastatic potential and are associated with poor prognosis [[33], [34], [35]]. Ca_1_Mab-3 and Ca_1_Mab-5 might be able to capture CTCs or clustered CTCs efficiently.

CDH1 functions as a tumor suppressor, and the loss of CDH1 plays a central role in tumor metastasis [36]. The downregulation of CDH1 during EMT has traditionally been associated with an enhanced metastatic potential by enabling cancer cell dissociation and invasion [8,37]. Nevertheless, the notion that the loss of CDH1 universally drives metastasis is an oversimplification, as numerous metastatic lesions retain high CDH1 expression, and epithelial cancer cells can acquire invasive and metastatic capacities without a complete EMT [38,39]. Notably, among breast cancer subtypes, loss of CDH1 expression is characteristic only of lobular carcinoma, whereas the majority of ductal carcinomas and their metastases retain CDH1 expression [40,41]. Furthermore, clustered CTCs in mammary tumors demonstrate greater metastatic efficiency than single disseminated tumor cells, with CDH1-mediated collective migratory behaviors that promote invasion and dissemination [42,43]. The phenomenon is explained by the partial EMT which defined by incomplete loss of epithelial markers and incomplete gain of mesenchymal markers [44]. The partial EMT plays a critical role in not only tumor invasion/metastasis, but also refractoriness to treatment [45]. More recently, a pro-metastatic role of CDH1 has also been proposed, attributed to its capacity to enhance cancer cell survival [46]. An anti-mouse CDH1 mAb reduced lung metastasis from genetically modified MMTV-PyMT mammary tumors or orthotopically grafted 4T1 tumors [47]. Therefore, it is worthwhile to capture tumor cells with partial EMT by Ca_1_Mabs and evaluate the anti-metastatic ability *in vivo*.

We converted the isotype of mAbs into mouse IgG_2a_ to obtain antibody-dependent cellular cytotoxicity (ADCC). These mAbs were mainly used for evaluating antitumor activities in mouse xenograft models [48,49]. Since the subclass of Ca_1_Mab-3 or Ca_1_Mab-5 is mouse IgG_1_, it does not exert ADCC. A class-switched Ca_1_Mab-3 or Ca_1_Mab-5 will be helpful to investigate the effect of tumor metastasis in mouse models. Additionally, both Ca_1_Mab-3 and Ca_1_Mab-5 recognize the cell-cell junction in IHC in normal squamous epithelium (Fig. 8C). Therefore, the side effects on normal epithelial cells should be investigated by Ca_1_Mab-3 and Ca_1_Mab-5. It is possible to evaluate whether the ADCC-mediated cell lysis is induced against normal epithelial cells by Ca_1_Mab-3 or Ca_1_Mab-5. If Ca_1_Mab-3 and Ca_1_Mab-5 can recognize mouse CDH1, the side effects will be evaluated in mice.

## Author disclosure statement

The authors have no conflict of interest.

## Funding information

This research was supported in part by 10.13039/100009619Japan Agency for Medical Research and Development (10.13039/100009619AMED) under Grant Numbers: JP25am0521010 (to Y.K.), JP25ama121008 (to Y.K.), JP25ama221153 (to Y.K.), JP25ama221339 (to Y.K.), and JP25bm1123027 (to Y.K.), and by the 10.13039/501100001691Japan Society for the Promotion of Science (10.13039/501100001691JSPS) Grants-in-Aid for Scientific Research (10.13039/501100001691KAKENHI) grant no. 25K10553 (to Y.K.).

## CRediT authorship contribution statement

**Rena Ubukata:** Investigation. **Hiroyuki Suzuki:** Writing – original draft. **Mika K. Kaneko:** Conceptualization. **Yukinari Kato:** Conceptualization, Funding acquisition, Project administration, Writing – review & editing.

## Declaration of competing interest

The authors declare that they have no known competing financial interests or personal relationships that could have appeared to influence the work reported in this paper.

## Data Availability

Data will be made available on request.
